# Design and implementation of a data sharing API for supporting urban governance schemes in environmental and traffic monitoring

**DOI:** 10.1016/j.mex.2025.103458

**Published:** 2025-07-05

**Authors:** Lasith Niroshan, Sarbast Moslem, Francesco Pilla

**Affiliations:** a School of Architecture Planning and Environmental Policy, University College of Dublin, Belfield, Dublin, Ireland; b Department of Civil, Structural and Environmental Engineering, Trinity College Dublin, the University of Dublin, Ireland; c Research Center of Digital Economics, Azerbaijan State University of Economics (UNEC), Istiglaliyyat Str. 6, Baku, Azerbaijan

**Keywords:** Software design, Geographic information system, urban governance, Data Sharing API, Environmental Monitoring, A data-sharing API design for Urban Governance

## Abstract

This paper presents the design and implementation of a novel, unified data-sharing API that integrates real-time air quality, noise, and traffic monitoring data to support urban planning and governance. Unlike existing API frameworks for urban data management that often focus on static or delayed datasets, our API uniquely integrates multi-source data with real-time processing capabilities. While prior research often addresses environmental monitoring or traffic analytics separately, our approach provides a single, comprehensive platform, enhancing usability for urban planners. The API provides a unified platform for collecting, processing, and sharing environmental and traffic data from diverse sources, including sensors for air quality, noise levels, and traffic conditions. The system facilitates real-time data integration, enabling urban planners, transport authorities, and policymakers to make informed decisions based on accurate environmental and traffic data. The API supports four key governance schemes in urban planning: User Demand Planning, Transport Planning, Freight and logistics Planning, and City Infrastructure Planning. By integrating environmental and traffic monitoring through a robust API architecture, this project connects technical innovation with real-world applications, supporting smarter urban management. This paper discusses the architecture, functionality, and impact of the API, demonstrating its ability to enhance data-driven decision-making for sustainable and efficient cities.

• Developed a unified API that integrates real-time air quality, noise, and traffic data from diverse sensors to support comprehensive urban data-sharing.
• Enables four key urban governance schemes—User Demand, Transport, Freight and logistics, and City Infrastructure Planning—through accurate, integrated environmental and traffic data.
• Bridges technical innovation and practical application, enhancing data-driven decision-making for sustainable, efficient urban management.

## Graphical abstract

**Figure:**
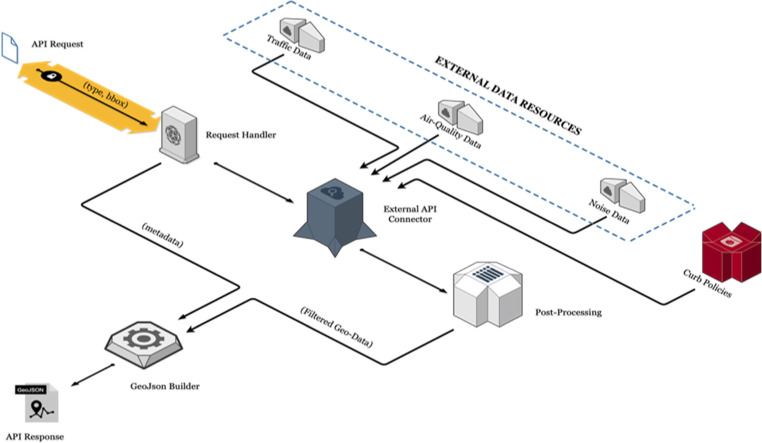

**Specifications table**

**Table:** 

**Subject area**	Computer Science
**More specific subject area**	*API Architecture Design*
**Name of your method**	*A data-sharing API design for Urban Governance*
**Name and reference of the original method**	*None*
**Resource availability**	Traffic signals and SCATS sites locations DCC: https://data.gov.ie/dataset/traffic-signals-and-scats-sites-locations-dcc *Noise and Air Quality Monitoring API DCC - Sonitus API:*https://data.smartdublin.ie/dataset/sonitus/resource/18befa04–7934–4ac8-b68d-5eeb7e76493d *Zaragoza’s traffic map:*https://www.zaragoza.es/sede/portal/movilidad/trafico/

## Background

Urban areas are complex, interrelated networks where infrastructure, transport, and environmental elements together influence the living standards of inhabitants [1]. These components do not operate in isolation; rather, they engage dynamically, producing ripple effects that affect urban livability and sustainability [2]. As urban areas expand and evolve, the demand for coordinated monitoring systems that tackle essential factors such as air quality, noise disturbances, and traffic behavior becomes progressively vital [3]. Grasping these interactions is crucial for developing cities that are efficient, equitable, and environmentally sustainable. Among these elements, traffic observation stands out as a fundamental aspect of urban science. Traffic encompasses more than just cars on highways—it is a complex system affecting transportation effectiveness, ecological well-being, and community health. Mismanaged traffic intensifies congestion, deteriorates air quality, and increases noise pollution, all of which hinder the objectives of sustainable urban development. Through the integration of real-time traffic information into city management, planners can develop flexible approaches to tackle these issues [4]. For example, improved public transit routes, flexible traffic control systems, and sustainable city planning can greatly lessen the negative effects of congestion [5].

At the core of urban functionality is logistics, which directly influences economic vitality and environmental sustainability [6]. Effective logistics systems rely significantly on comprehending the complex interactions among freight transport, traffic trends, and environmental limitations. Traffic surveillance, an essential component of logistics, allows for adaptive routing, congestion control, and delivery enhancement [7]. These features reduce delays, improve supply chain dependability, and guarantee that logistics support wider urban sustainability goals.

Nonetheless, combining environmental data with traffic information introduces an extra level of innovation. For instance, live air quality and noise data can guide logistical choices, such as redirecting large vehicles from pollution-sensitive regions or areas with many pedestrians, thus minimizing the environmental impact of freight transport [8,9]. Other urban monitoring initiatives (OpenAQ for air quality and traffic APIs) have made progress in exposing domain-specific data, yet they often lack cross-domain integration or governance-oriented alignment. Our API addresses this gap by unifying real-time environmental and traffic data streams to support direct decision-making in key urban planning contexts.

Recent work by Bokirci, demonstrates the value of high-resolution, real-time environmental monitoring using UAVs for city-scale air quality mapping [10]. Their approach addresses spatial gaps in fixed monitoring systems, underscoring the growing need for flexible and adaptive sensing platforms. This complements our fixed-sensor API by highlighting the importance of scalable, real-time data infrastructures in smart urban governance.

This paper presents a reliable data-sharing API that combines environmental and traffic monitoring information to aid urban planning and governance. The API aims to simplify the gathering, processing, and distribution of data from various sources, such as air quality sensors, noise detectors, and traffic monitoring systems. It is designed to focus on four essential governance frameworks in urban planning: User Demand Planning, Transport Planning, Freight & Logistics Planning, and City Infrastructure Planning. Through real-time data integration and analysis, the API equips decision-makers with actionable insights to improve urban efficiency, sustainability, and livability. The API represents optimal methods in data integration and analysis, converting raw data into a valuable asset. Its design is intended for scalability and flexibility, effortlessly handling various datasets while enabling sophisticated analytics features like predictive modeling and scenario simulations. The API enables flexible approaches for user demand planning, transportation optimization, freight logistics, and urban infrastructure enhancement. Doing this improves urban areas' resilience and sustainability, enabling cities to address the demands of contemporary urbanization while emphasizing livability and care for the environment.

## Method details

**Fig. 1:**
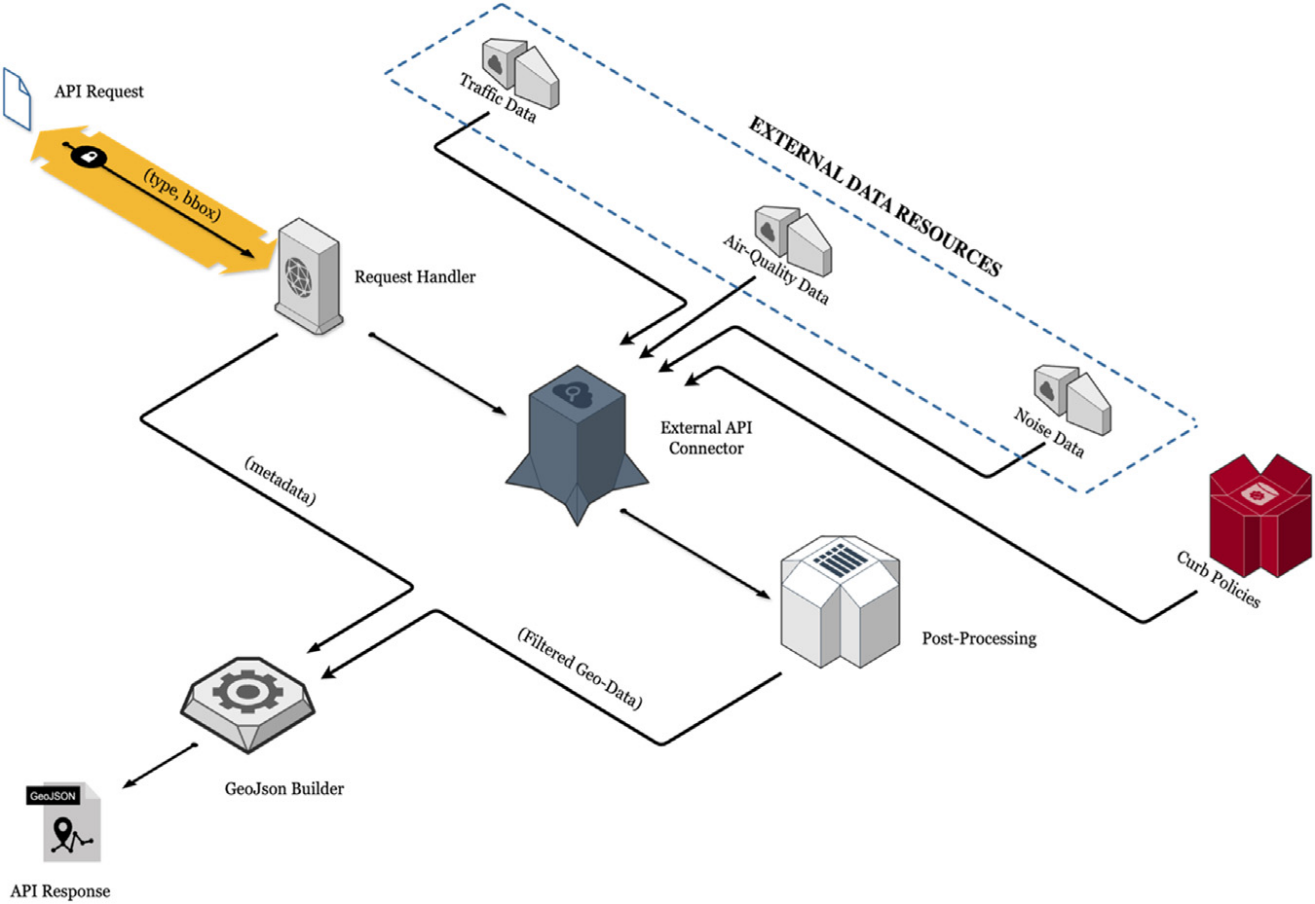
High-level architecture of the data-sharing API. The system integrates diverse environmental and traffic monitoring data sources, including air quality sensors, traffic sensors, and spatial datasets (e.g., curb zones).

The development of the data-sharing API centers on providing a robust, scalable platform to integrate diverse environmental and traffic monitoring datasets. The architecture leverages modern web technologies and geospatial standards to support urban governance schemes through seamless data exchange and interoperability. Fig. 1 illustrates the system's high-level architecture, detailing its core components and their interactions.

### Data sources

To effectively support urban governance schemes and environmental monitoring, the API integrates a diverse range of data sources, including traffic data, air quality data, noise data, and curb policy information. These datasets are obtained from both official repositories and sensor networks. The use of official data sources ensures high data reliability and accuracy, which is critical given the socio-sensitive nature of urban planning and decision-making. Additionally, sensor-based data is incorporated in a controlled and verified manner to maintain credibility and trustworthiness. By combining these sources, the API provides a robust foundation for data-driven governance and planning decisions.

#### Curb policy information

Curb policy information plays an essential role in urban management by regulating the use of curbside spaces. This research leverages three key datasets to provide comprehensive details of curbside regulations. The following table summarizes the datasets used: (Table 1).

**Table 1:** Datasets used in Curb Policy extraction.

*Dataset*	*Description*	*Key Attribute*
*Curb Policies*	*Defines rules and regulations for curb usage, including parking and time limits.*	*Policy ID, Zone ID, Permitted Activities, Time Limits*
*Curb Zones*	*Spatial data representing curb zones with their characteristics.*	*Zone Type, Location, Operational Hours, Dimensions*
*Loading Zones*	*Specific data for areas designated for loading/unloading activities.*	*Coordinates, Length, Width, Loading Times*

These datasets are aggregated and filtered within the API to ensure users receive accurate and context-specific information. The API allows spatial queries, such as bounding box (bbox) filtering, to retrieve relevant curb policy details. This functionality supports urban planners and policymakers in optimizing curb space allocation and improving traffic management strategies.

#### Traffic data

Since the experiments were based in Dublin, Ireland, and Zaragoza, Spain, the traffic data sources and formats were different, and getting a unified output was challenging. This section explores the diversity of the two datasets.

1. Traffic data from Zaragoza

**Fig. 2:**
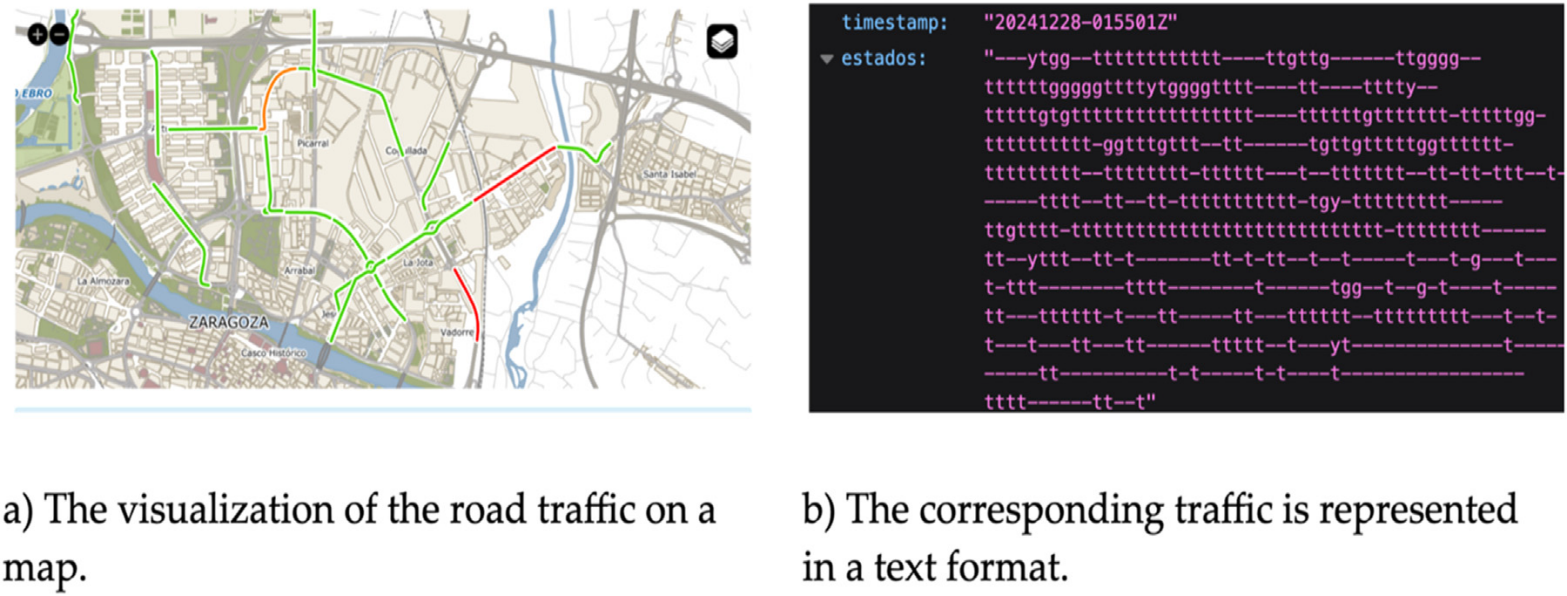
The traffic data visualization and API output.

Zaragoza’s traffic map^1^ Visualizes real-time traffic for monitored roads. There is also an API endpoint to get real-time traffic data in text format. The “Estados” field contains a string in which each character represents the status of a monitored road according to the position it occupies in the string. A separate endpoint provides the coordinates of the monitored roads. (Fig. 2).

2. Traffic data from Dublin

The traffic data for Dublin, provided by the Dublin City Council (DCC), forms a critical part of this study (Dublin City Council, 2023). The dataset includes detailed information about the locations of junctions, pedestrian crossings with traffic lights, and SCATS (Sydney Coordinated Adaptive Traffic System) sites within the administrative area of Dublin City Council.

For our experiments, we specifically utilized the SCATS dataset, which offers a robust foundation for analyzing and optimizing urban traffic flow. SCATS is an adaptive urban traffic management system designed to synchronize traffic signals, improving traffic efficiency across cities, regions, or corridors. Table 2 presents the attributes of the dataset.

**Table 2:** Attributes of the SCATS dataset provided by Dublin City Council.

Attribute	Description
SiteID	Unique identifier for a signal or SCATS site.
Site_Description_Cap	Site description in capital letters.
Site_Description_Lower	Site description in lowercase letters.
Region	Refers to the SCATS regional servers.
Lat	Geographic location (latitude).
Long	Geographic location (longitude).
Site_Type	The type of site indicates whether it is a SCATS site or a signal site.

#### Air quality data

Air quality data plays a key role in understanding environmental conditions and their impact on urban governance schemes. For this study, air quality data were sourced from official providers in Zaragoza, Spain, and Dublin, Ireland. This section outlines the datasets, endpoints, and data attributes used for analysis.

1. Air Quality Data from Zaragoza

The Zaragoza City Council provides air quality data through a dedicated API endpoint. This API delivers real-time readings from a network of air quality monitors distributed across the city. The data includes measurements of particulate matter, gaseous pollutants, and associated metadata.

2. *Air Quality Data from Dublin*

In this study, air quality data for Dublin was sourced from the SONITUS API [11], which provides real-time and historical environmental data. The SONITUS API supports multiple endpoints, including retrieving the latest readings and average readings over specific periods, monitoring metadata, and monitoring types. These endpoints enable the efficient collection of air quality metrics such as PM10, PM2.5, NO2, and CO, along with associated metadata like timestamps and monitor locations. This robust dataset supports detailed spatial and temporal analyses of air quality conditions across Dublin.

### Noise data

The Sonitus API is used to fetch real-time noise data, which is hosted by Dublin City Council [11]. This API forms part of the national air quality monitoring network, providing continuous and accurate insights into environmental noise levels. Access to the Sonitus API is secured through a username and password, which users must obtain before use. Unlike other APIs, the Sonitus API is designed to return data for a single monitoring station per request, which necessitates multiple calls when data from multiple sensors are required.

#### The request handler

Once the SENATOR service (e.g., Front-End or a third-party request) submits a request for specific data, the Request Handler becomes the primary module responsible for managing the interaction. It processes, validates, and routes user requests efficiently, ensuring the system's integrity and optimal performance. This module ensures that only valid requests are passed along the data pipeline, maintaining the system's integrity and performance.

The Request Handler performs the following key operations:

1. Request Validation: It verifies the structure and parameters of incoming requests to ensure compliance with the API's requirements. Invalid or malformed requests are rejected with appropriate error messages.
2. Bounding Box Filtering: For spatial queries, the Request Handler extracts the bbox parameters from the request. It uses these parameters to filter data geographically, ensuring that only data within the specified box is retrieved. If the external API lacks built-in geospatial filtering capabilities, this filtering is instead performed during the post-processing stage.
3. Data Type Selection: The Request Handler identifies the requested data type (e.g., traffic, air quality, noise, or curb policy data) from the input parameters. This selection streamlines the data retrieval process by directing the query to the relevant data source.
4. Routing to the External API Connector: Once the request is validated and pre-processed, the Request Handler forwards it to the External API Connector. The Connector then communicates with the respective external systems to retrieve the requested data.

By efficiently managing these tasks, the Request Handler ensures that the system operates smoothly, providing users with accurate and relevant information while optimizing resource usage.

#### The external API connector

The External API Connector serves as a bridge between the Request Handler and external data providers and static databases. It is responsible for managing communication with pre-defined (explained in section 2.1) APIs to retrieve the requested data based on the filtered parameters. This modular design ensures efficient integration and interoperability with diverse external systems, enabling seamless data acquisition for urban governance and environmental monitoring.

*Below is a list of the main responsibilities of the external API connector.*

1. API Endpoint Communication

The Connector communicates with multiple APIs, including traffic, air quality, noise, and curb policy data sources. Each API has its structure, authentication requirements, and response formats, which the Connector handles efficiently.

2. Static Database Integration

In addition to real-time data from external APIs, the Connector extracts static datasets such as Curb Policy Information. This includes:

a. Curb Policies: Rules and regulations for curb usage, including parking restrictions and time limits.
b. Curb Zones: Spatial representation of curb zones with their operational attributes.
c. Loading Zones: Specific data for designated loading/unloading areas.

These static datasets are stored in a database and are accessed directly by the External API Connector to supplement the dynamic data from external APIs.

3. Dynamic Endpoint Mapping

The Connector dynamically maps the filtered parameters (e.g., bounding box, data type) from the Request Handler to the appropriate external API endpoints. For instance:

a. Traffic data for Dublin is retrieved via Dublin City Council’s SCATS API.
b. Air quality data for Zaragoza is fetched using the city council’s dedicated air quality monitoring API.
c. Noise data is obtained via the Sonitus API, managing authentication and station-specific requests.

4. Authentication and Authorization

Many external APIs, such as the Sonitus API, require authentication credentials. The Connector securely handles API keys, usernames, and passwords to ensure authorized access to data.

5. Response Parsing and Formatting

After fetching data from external APIs or static databases, the Connector parses the responses, reformats them into a standardized structure, and sends them to the data filtering module. This step is important for a consistent analysis, as each source may provide data in diverse formats.

6. Error Handling and Retry Mechanisms

The Connector includes robust error-handling mechanisms to address issues such as network failures, authentication errors, or invalid requests. In cases of temporary failures, it implements a retry mechanism to ensure successful data retrieval.

#### Post-Processing

The Post-Processing module refines and enhances the raw data retrieved from external APIs and static databases. This step ensures that the data meets the requirements for analysis, visualization, and integration into urban governance frameworks. The following post-processing procedures have been implemented:

1. Geospatial Filtering

Many API outputs are not inherently filtered by the bbox parameter provided during the request. To address this, the module applies a geospatial filter as the first step. This involves verifying that the retrieved data falls within the specified geographical boundaries and discarding irrelevant records outside the bbox.

2. Traffic Data String to Status Conversion

Specific traffic APIs, such as Zaragoza’s, represent traffic conditions as encoded strings where each character corresponds to the status of a monitored road. The module decodes this string into meaningful traffic statuses for each road segment.

3. *Error Handling*

The post-processing module incorporates robust error-handling mechanisms to identify and manage inconsistencies or anomalies in the data. For example, it flags missing fields and unexpected formats, ensuring that only reliable data progresses to subsequent stages.

4. *Filtering Out Null Values*

Null values in datasets can distort analyses and visualizations. The post-processing module filters out incomplete or invalid records, maintaining the integrity of the dataset. This step ensures that downstream applications, such as visualizations or decision-support tools, are based on high-quality data.

5. *Coordinate Conventions*

In some cases, the data sources use different coordinate systems or formats (e.g., decimal degrees, degrees-minutes-seconds). If necessary, the module standardizes all spatial data into a unified coordinate convention (EPSG:4326), ensuring compatibility across datasets and applications.

By implementing these post-processing procedures, the system guarantees that the data is not only accurate and relevant but also ready for immediate use in urban planning and environmental monitoring applications. This comprehensive approach enhances the reliability of analyses and supports informed decision-making.

#### Geo-JSON builder

The Data Sharing API generates a Geo-JSON output from the post-processed data as the final step. Geo-JSON is a widely used format for encoding geographic data structures and is particularly suitable for web applications and APIs [12]. The process begins by taking the cleaned and standardized spatial data and converting it into the Geo-JSON format, including features such as points, lines, polygons, and associated attributes.

The conversion process ensures that each dataset is represented with its respective geometry and properties in the standardized EPSG:4326 coordinate system, facilitating interoperability across applications. The resulting Geo-JSON file is structured to include metadata, feature IDs, and relevant information (e.g., air quality levels, traffic flow, noise levels, and curb policies) for each geographic feature.

However, converting heterogeneous spatial datasets into Geo-JSON presents several challenges. These include inconsistencies in coordinate reference systems (CRS), geometry validation errors (e.g., self-intersecting polygons), large file sizes that impact performance, and variations in attribute schemas across data sources. To address these, the API implements CRS normalization, geometry validation checks, and attribute harmonization before serialization. Compression mechanisms and pagination are also used when serving large Geo-JSON files to optimize performance and reduce client-side load times.

This Geo-JSON output is then served through the API, allowing it to be easily consumed by web applications, data visualization tools, or further processing pipelines. The format provides a flexible and extensible way to represent and share geospatial data, supporting effective decision-making for urban governance schemes.

#### API endpoint overview

The developed data-sharing API provides a set of endpoints designed for efficient querying and data sharing to facilitate the integration and retrieval of diverse datasets. These endpoints cover various aspects, including air quality, traffic, noise, and curb policies. Each endpoint supports specific parameters to allow for flexible and targeted data retrieval, ensuring compatibility with geospatial applications. The following table provides an overview of the implemented API endpoints, including their HTTP methods, parameters, and functionality (Table 3).

**Table 3:** An overview of the API endpoints.

HTTP Method	Endpoint	Parameters	Description
GET	/test	None	A simple test endpoint to check if the API is running.
GET	/appyway/search	key (required), value (required)	Searches AppyWay data using a key-value pair.
GET	/appyway/bbox_search	bbox (required)	Searches AppyWay data within a specified bbox.
GET	/curb/bbox_search	bbox (required)	Searches all datasets (AppyWay, loading zones, and curb zones) within a bbox and returns a combined result.
GET	/traffic/Zaragoza	bbox (required)	Retrieves traffic data from Zaragoza, filtered by a bbox.
GET	/air_quality/Dublin	bbox (required)	Retrieves the latest air quality readings from the EPA, filtered by a bbox.
GET	/air_quality/zaragoza	bbox (required)	Fetches air quality data from Zaragoza stations in a specified bbox.
GET	/noise_data_average/Dublin	bbox (required), hourly (optional)	Fetches hourly averages for noise data in a given bounding box.
GET	/noise_data/Dublin	bbox (required)	Retrieves real-time noise data from monitors in a given bbox.

This API architecture ensures that the data is easily accessible and adaptable to a wide range of environmental and urban planning applications. The endpoints are optimized for the performance of the front-end services and allow for seamless integration into existing workflows.

## Method validation

The experimental evaluation of the data-sharing API focuses on both its functional capabilities and its technical performance in supporting urban governance and environmental monitoring. This section presents the outcomes of the API's performance in integrating and delivering data across four key domains: curb policies, traffic, air quality, and noise. In addition to evaluating the utility of the API in enabling data-driven decision-making, we also conducted a detailed API performance evaluation. This involved benchmarking response times, throughput, and system reliability under load to validate the API’s readiness for real-world deployment. By presenting both the functional outcomes and performance metrics, this section demonstrates the API’s ability to provide scalable, accurate, and consistent access to heterogeneous urban data for stakeholders involved in planning and policy development.

### API performance evaluation

**Fig. 3:**
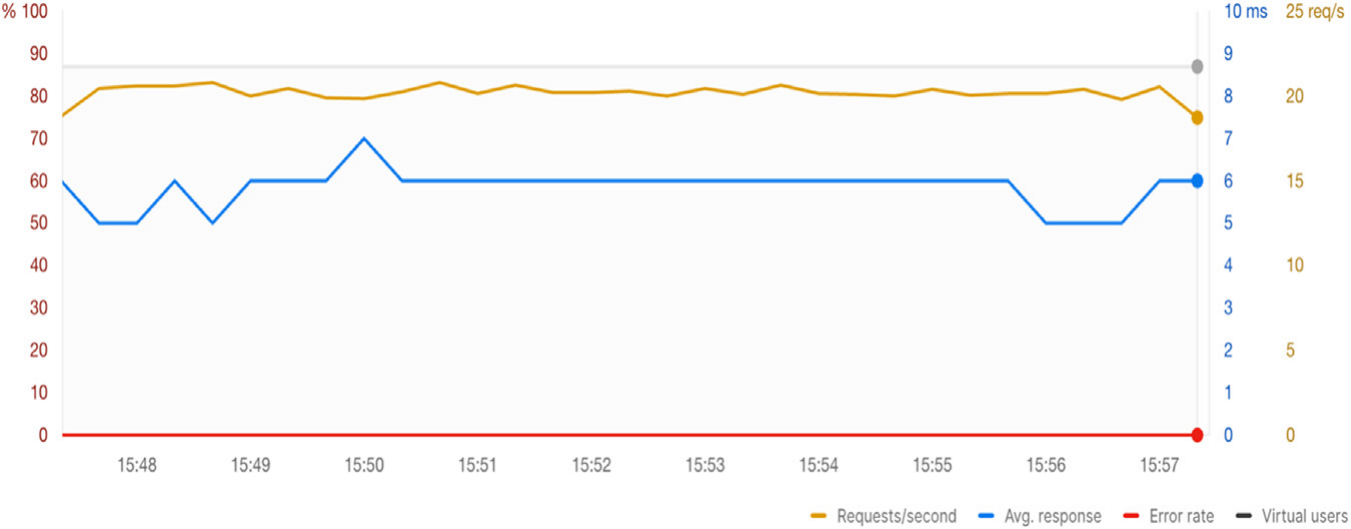
Stress testing and throughput analysis using Postman’s Runner.

To evaluate the performance of the Data Sharing API under simulated load conditions, we used Postman's Runner to issue a total of 12,246 requests to various endpoints within 10 min. The API demonstrated high responsiveness, with an average response time of 6 ms and zero error rate (0.00 %) throughout the test. The system sustained a throughput of approximately 20.22 requests per second, indicating the backend can handle moderate traffic efficiently. Latency percentiles further confirm performance stability, with P90 at 8 ms, P95 at 9 ms, and P99 at 14 ms, showing minimal variance under load. These results validate the system's ability to maintain low-latency communication and robust reliability in scenarios aligned with small to mid-sized deployments. A graphical summary of the response times and throughput distribution is provided in Fig. 3.

### Retrieval of curb policies via API

**Fig. 4:**
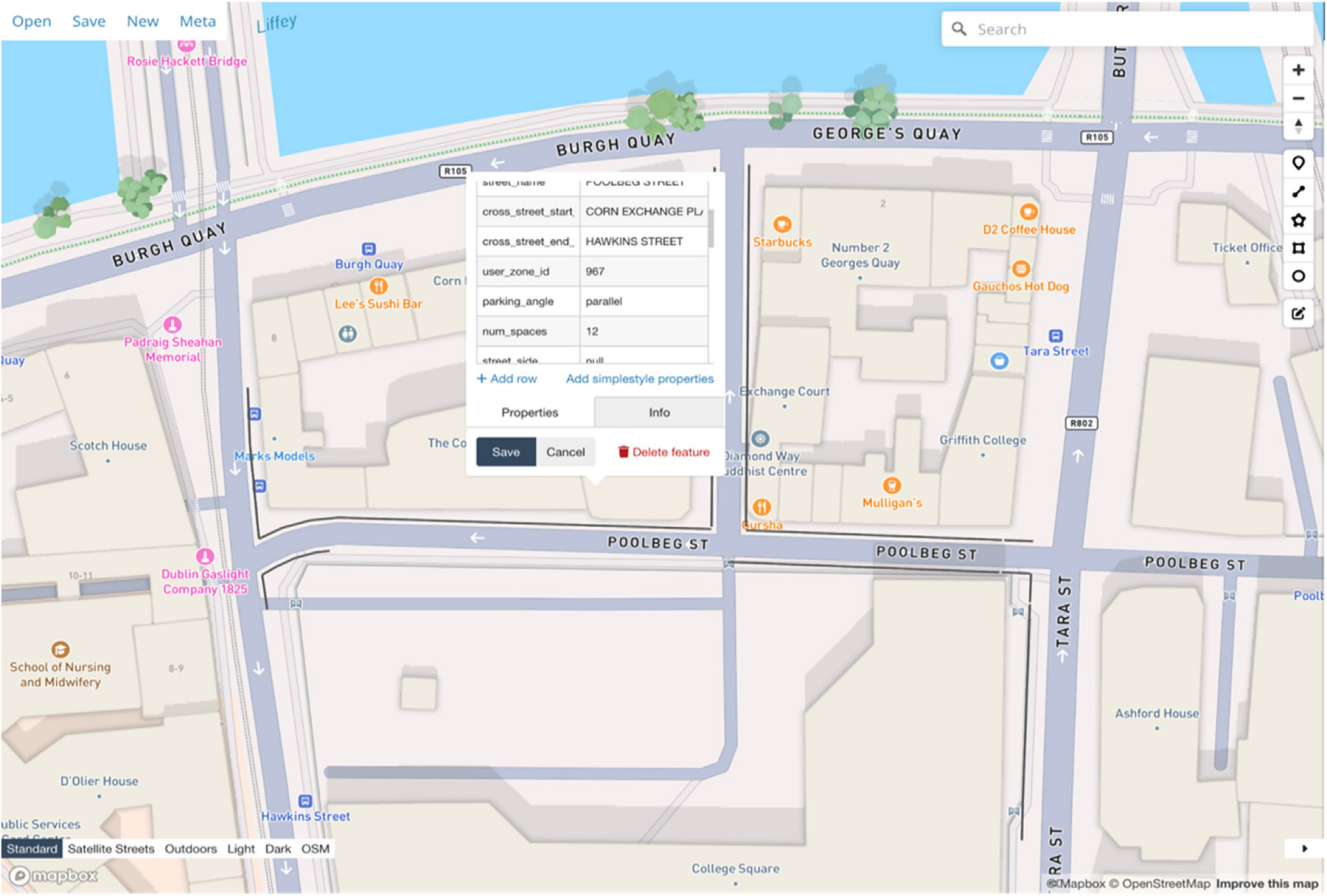
Visualization of the API output using the GeoJSON.io application.

The first experimental result evaluates the retrieval of curb policy data through the /curb/bbox_search endpoint. This API query enables users to access detailed curb-related information within a specified bbox. The retrieved data is structured as a GeoJSON object, facilitating seamless integration with geospatial tools for visualization and analysis (See Fig. 4).

The dataset provides rich details, including spatial representations of curb zones as line strings, regulation types such as “No Parking” or “Undesignated,” and additional attributes such as number of bays available and priority levels. Temporal information, such as specific times, days, and months when the regulations apply, is also included. Front-end visualization applications can use this data to depict regulated curb areas on a map, with distinct color coding to represent different regulation types and priorities.

While this endpoint successfully retrieves curb policy data within the specified bounding box, a notable limitation is its lack of clipping functionality. As a result, road segments extending beyond the requested box are included in their entirety. This limitation can introduce minor inaccuracies in the spatial representation of curb data, which will be discussed further in the subsequent sections.

### Retrieval of traffic data via API

The retrieval of traffic data was evaluated using the /traffic/Zaragoza endpoint, which provides real-time traffic conditions for the monitored roads in Zaragoza. This endpoint accepts a bounding box parameter, enabling users to focus on specific geographic areas. The data is returned in GeoJSON format, allowing for seamless integration with geospatial visualization tools and third-party applications.

The API leverages two primary datasets: the Zaragoza Traffic Map and the Traffic Monitors Dataset. The Traffic Map encodes traffic conditions as a string, with each character representing the state of a monitored road segment. The encoding structure is summarized in the table below: (Table 4).

**Table 4:** Traffic condition encoding structure used in the Zaragoza City Council Traffic Map API.

Character	Description	Meaning
*R*	*Red*	*High congestion*
*Y*	*Yellow*	*Moderate congestion*
*G*	*Green*	*Smooth traffic*
*B*	*Black*	*Road closure or blockage*
*T*	*Transparent*	*Unknown or unmonitored*
*–*	*N/A*	*No data available*

**Fig. 5:**
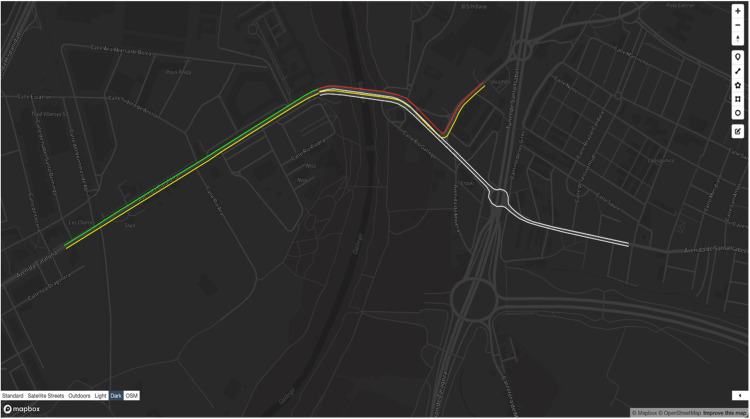
Visualization of traffic data processed by the API.

The Monitors Dataset provides the spatial coordinates of the monitored road segments, allowing the API to associate traffic conditions with their respective locations. The API decodes the traffic string into meaningful statuses through post-processing and maps them to corresponding road segments. The output visualization highlights traffic density across the specified area while color coding to represent different levels of congestion. Fig. 5 illustrates a sample output using a third-party visualization application.

### Retrieval of air quality data via API

**Fig. 6:**
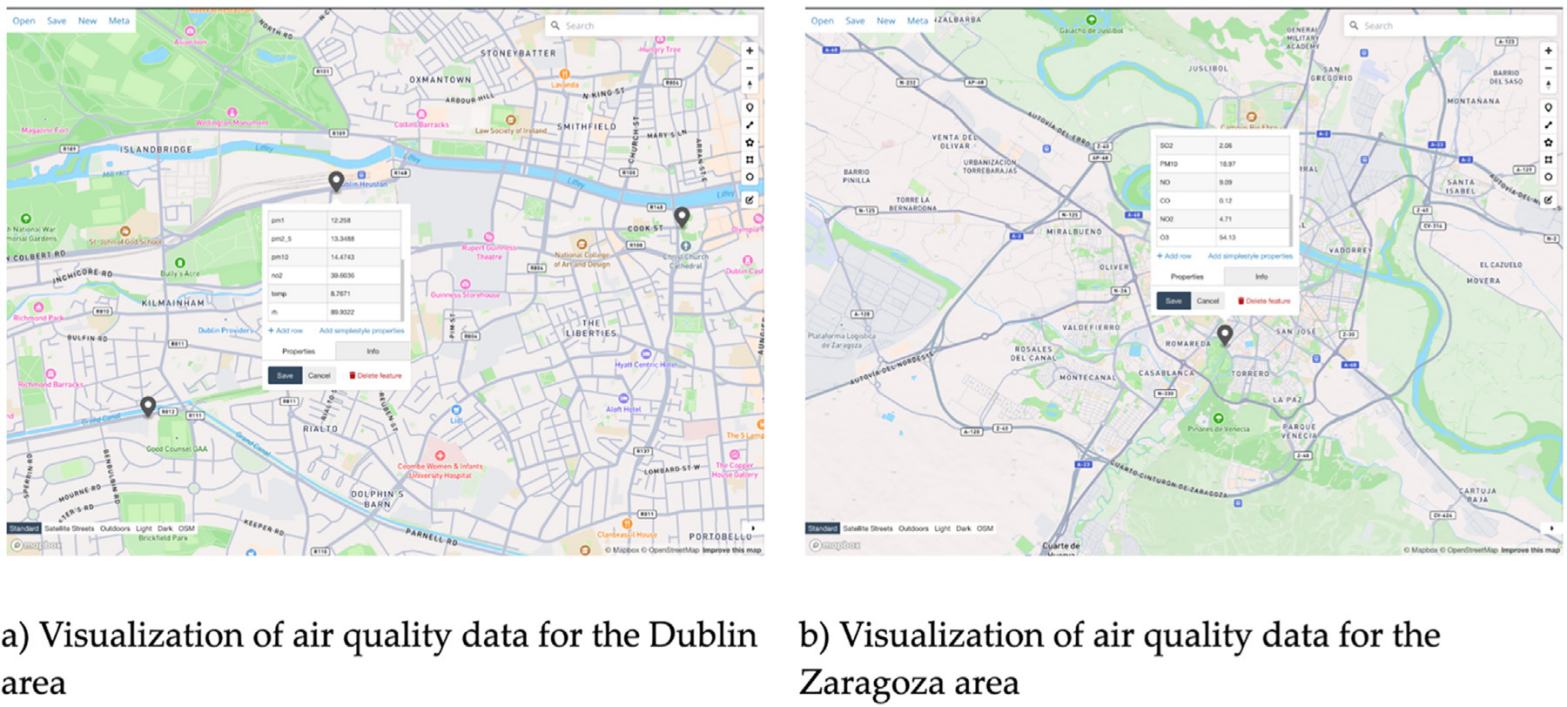
Sample visualizations of API output for air quality endpoints.

The retrieval of air quality data was evaluated using the /air_quality/Dublin and /air_quality/Zaragoza endpoints, designed to provide real-time and historical air quality metrics for specific geographical areas. Both endpoints accept a bounding box parameter, enabling users to query data for a defined spatial extent. The data is returned in GeoJSON format, making it compatible with geospatial visualization and analysis tools. The following image visualizes sample outputs from two API endpoints for Dublin and Zaragoza, respectively (Fig. 6).

The data-sharing API returns a unified output, including measurements for pollutants (e.g., NO2, SO2, CO, O3) and particulate matter (PM10 and PM2.5). Additional metadata, such as timestamps and sensor locations, is included to provide temporal and spatial context for the readings. While the endpoints effectively retrieve city-specific data, their polymorphic nature (/air_quality/Dublin and /air_quality/Zaragoza) raises the possibility of duplication. Although this design simplifies task-specific queries, a future improvement could involve consolidating these endpoints into a single endpoint, with geo-context switching handled at the backend.

### Retrieval of noise data via API

The retrieval of noise data was evaluated using the /noise_data/Dublin endpoint, which integrates data from the Sonitus API hosted by the Dublin City Council. The Sonitus API forms part of the national air quality monitoring network, providing continuous, real-time insights into environmental noise levels. Noise data is retrieved from monitoring stations strategically deployed across Dublin at a high temporal resolution.

The API outputs noise levels in decibels (dB) along with metadata such as timestamps and sensor locations. This detailed dataset is well-suited for spatial and temporal analyses, enabling the identification of noise pollution hotspots in urban areas. However, as the Sonitus API is designed to return data for a single monitoring station per request, multiple API calls are required to retrieve data from all sensors within a specified bounding box. The noise data can be used to identify areas with high noise levels, such as busy intersections, commercial areas, and transport hubs. This provides valuable insights for urban planners to reduce noise pollution.

At present, noise data is only available for Dublin through this API. Incorporating noise data for Zaragoza is planned as a future enhancement to the data-sharing system, which will allow for cross-city analyses and further strengthen the API's capabilities.

## Limitations

The proposed data-sharing API successfully integrates environmental and traffic monitoring data, facilitating real-time decision-making in urban governance. The system's ability to support four governance schemes—user Demand Planning, Transport Planning, Freight and logistics Planning, and City Infrastructure Planning—demonstrates its applicability in urban planning scenarios. The API's structure enables efficient data retrieval while maintaining modularity and scalability for smart city applications.

Compared to existing API frameworks for urban data management, our API uniquely integrates multi-source data with real-time processing capabilities. While prior research focuses on environmental monitoring or traffic analytics separately, our approach integrates both into a single platform, enhancing usability for urban planners. The hybrid RESTful and edge-computing-based design also provides flexibility, a feature not commonly explored in previous implementations.

A monolithic API architecture was deliberately chosen for this initial, minimum viable product (MVP) implementation to simplify deployment and streamline system management during development and testing. Given the relatively modest scale of the current deployments (Dublin and Zaragoza), the monolithic approach allowed for faster iteration and debugging. However, we acknowledge that a microservices-based architecture could offer advantages in terms of fault isolation, independent scalability, and maintainability as the system expands. Accordingly, future versions will transition to a microservices architecture orchestrated using Kubernetes, allowing for modular, resilient, and dynamically scalable service deployment.

Future improvements include extending the API to additional cities beyond Dublin and Zaragoza, ensuring adaptability to diverse urban contexts. Moreover, implementing a location-agnostic architecture, where users can dynamically specify the geographic context rather than relying on city-specific endpoints, will enhance the API's scalability and reduce potential duplication.

While the API effectively integrates environmental and traffic data, the security of the data continues to be an essential concern. It is important to protect data accuracy and individuals' privacy when handling sensitive urban data. Future versions will adopt OAuth 2.0 for federated identity management, allowing for secure and flexible authentication across multiple user roles, institutions, or municipal departments. Additionally, HTTPS will be enforced for all data transmissions, ensuring end-to-end encryption and safeguarding the API against common threats such as man-in-the-middle attacks. These enhancements will strengthen both user privacy and system resilience in real-world smart city deployments.

We acknowledge that the current use of city-specific endpoints (e.g., /air_quality/dublin, /air_quality/zaragoza) introduces redundancy and reduces scalability. This design choice was initially adopted for simplicity during MVP testing and deployments. However, future iterations of the API will adopt a generic, location-agnostic endpoint structure (e.g., /air_quality?city=Dublin) with backend geo-context switching. This approach will reduce code duplication, enhance maintainability, and allow dynamic support for multiple cities without modifying the endpoint structure. It also aligns better with RESTful principles and promotes extensibility as the system scales to new urban contexts.

Overall, this work lays a foundation for scalable, real-time urban data integration, bridging the gap between technical innovation and governance applications. Future refinements will focus on extending the API's reach and strengthening its security features.

## For a published article

*None*.

## Ethics statements

This study did not involve human subjects, animal experiments, or data collected from social media platforms. All data used in this research were obtained from publicly available sources and processed in compliance with ethical guidelines for geospatial data analysis. The study adheres to responsible data usage principles, ensuring that no personally identifiable information (PII) was included. Additionally, the development and implementation of the API align with internal project protocols and institutional research ethics standards.

## Declaration of competing interest

The authors declare that they have no known competing financial interests or personal relationships that could have appeared to influence the work reported in this paper.

## Data Availability

The data that has been used is confidential.

## Footnotes

1 https://www.zaragoza.es/sede/portal/movilidad/trafico/.
